# Timely Albumin Infusion May Improve Resource Utilization in Patients with Cirrhosis and Spontaneous Bacterial Peritonitis

**DOI:** 10.1155/2024/6673823

**Published:** 2024-06-08

**Authors:** W. Ray Kim, Karthik Raghunathan, Greg S. Martin, E. Anne Davis, Navreet Sandhu Sindhwani, Santosh Telang, Kunal Lodaya

**Affiliations:** ^1^ Division of Gastroenterology and Hepatology Stanford University, Stanford, CA, USA; ^2^ Department of Anesthesiology Duke University, Durham, NC, USA; ^3^ Department of Medicine Emory University, Atlanta, GA, USA; ^4^ Grifols SSNA, Research Triangle Park, NC, USA; ^5^ Boston Strategic Partners Inc., Boston, MA, USA

## Abstract

Spontaneous bacterial peritonitis is a life-threatening complication of cirrhosis that can increase healthcare utilization. The impact of albumin administration timing on hospital resource utilization and its optimal timing is unclear, despite its efficacy in improving survival for cirrhosis patients with spontaneous bacterial peritonitis. A retrospective study was conducted to evaluate the influence of the timing of albumin administration on the length of stay and total hospital cost for patients with cirrhosis and spontaneous bacterial peritonitis who require fluid resuscitation. The study utilized de-identified data from Cerner Health Facts® data. Adult inpatients with a diagnosis of cirrhosis and SBP receiving ≥1 antibiotic and fluid resuscitation between January 1, 2009, and April 30, 2018, were included and stratified by albumin administration timing: ≤24 hours from hospital admission (“timely albumin”) or >24 hours of admission or no albumin (“non-timely albumin”). We used a Kaplan-Meier curve with log-rank test to evaluate the association between timing of albumin administration and time to hospital discharge and a generalized linear model to examine the association between albumin timing and total hospital costs. We identified 1,308 hospitalizations, of which 301 contained valid cost data. The timely albumin group had a median time to discharge of 6.95 days compared to 7.78 days in the non-timely group (*p* = 0.02). Cost model showed that receiving timely albumin incurred 16% lower costs (*p* = 0.027) than patients in the non-timely albumin group. Timely albumin administration with an antibiotic regimen may shorten the length of stay and lower costs, thereby reducing hospital resource utilization in patients with cirrhosis and spontaneous bacterial peritonitis requiring fluid resuscitation.

## 1. Introduction

Cirrhosis is a long-term consequence of cumulative, often irreversible, liver injury associated with increased risks of morbidity, mortality, and hospital admission and readmissions [1–4]. Among patients with cirrhosis and ascites, the occurrence of spontaneous bacterial peritonitis (SBP) demarcates a late phase of end-stage liver disease characterized by portal hypertension and splanchnic vasodilation, bacterial translocation, cytokine dysregulation, and hemodynamic perturbations [5, 6]. SBP accounts for at least 24% of the bacterial infections that occur in patients with cirrhosis and has a mortality rate ranging from 20% to 50% [7–11].

Patients with decompensated cirrhosis incur higher hospital costs, as end-stage liver complications such as SBP can drastically increase hospital length of stay (LOS) and resource utilization, ultimately placing a significant burden on healthcare systems [12–14]. The total annual cost of hospitalization in patients with cirrhosis and cirrhosis-related complications was recently reported to range from $7 billion to over $16 billion [15, 16], and a majority of the cost was attributable to decompensated cirrhosis [15].

The American Association for the Study of Liver Diseases' (AASLD) 2009 and 2012 guidelines on the management of SBP recommends albumin infusion as an important component in managing patients with end-stage liver disease experiencing complications including grade 3/refractory ascites, acute kidney injury (AKI), and hepatorenal syndrome [17, 18]. Previous studies have shown that albumin is associated with decreased mortality and improved patient outcomes among patients with cirrhosis and SBP [19–29]. However, optimal timing of albumin administration and its impact on healthcare utilization is not well understood. In this study, we used a nationwide electronic health record (EHR) database to identify patients with cirrhosis and SBP, who received fluid resuscitation and evaluated the association between timing of albumin administration and hospital resource utilization, measured by the length of hospital stay and the total cost of hospitalization.

## 2. Methods

### 2.1. Study Design/Group Selection

We utilized a nationwide, de-identified, HIPAA-compliant, EHR database (Cerner Health Facts^®^, Cerner Corp., Kansas City, MO) to extract real-world data from more than 700 participating clinical facilities and hospital systems across the United States. This observational study received Institutional Review Board exemption through federal regulation 45CFR46 by the Western Institutional Review Board (Puyallup, WA), based on a predefined statistical analysis plan (Ref. # 1-1137087-1; approved December 6, 2018).

**Figure 1:**
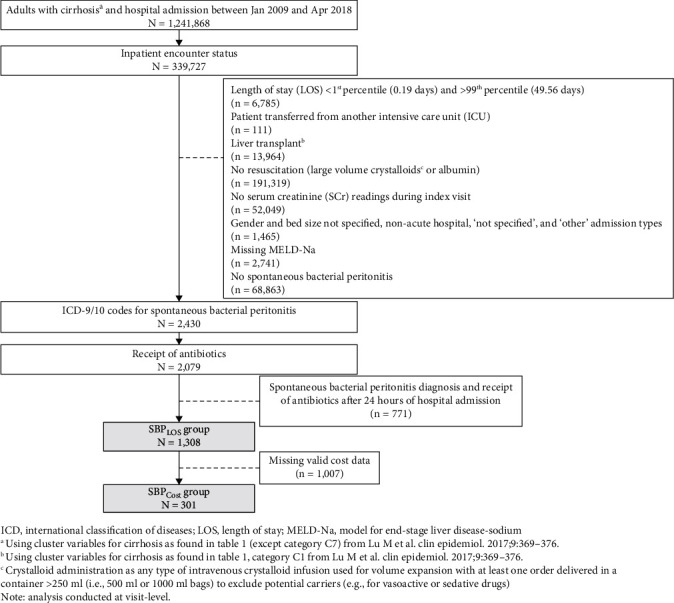
Patient flow selection diagram.

We identified inpatient hospital encounters between January 1, 2009, and April 30, 2018, where patients ≥ 18 years old were diagnosed with cirrhosis and SBP, received ≥1 antibiotic, and received fluid resuscitation during their stay. Fluid resuscitation was defined as any type of intravenous crystalloid infusion used for volume expansion with at least one order delivered in a container > 250 mL. Cirrhosis was identified using International Classification of Disease (ICD-9/10) codes and current procedural terminology codes, and SBP was identified using ICD-9/10 codes (Supplementary Table 1). Administration of antibiotics was identified according to the Veteran Affairs National Formulary list, which comprises all routes of administration (Supplementary Table 2). We defined SBP diagnosis based on the time of encounter admission in the dataset. This was further validated using time of hospital admission and receipt of antibiotics, where only patients with both an SBP diagnosis and receipt of antibiotics within 24 hours of admission were included in the final cohort. We also explored the occurrence of potential nosocomial SBP cases during hospitalization in our population using ICD-10-CM code Y95 that indicates a nosocomial condition. Additionally, multi-drug-resistant organism (MDRO) prevalence was identified using ICD-10-CM codes (Z16.X). Criteria for patient selection are presented in Figure 1.

We examined our study population for two key outcomes: hospital LOS measured by time to discharge from the hospital (SBP_LOS_) and total hospital costs. In the LOS analysis, we censored for death (i.e., patients discharged with a status of “deceased”) and assessed time to discharge using a Kaplan-Meier analysis. We assessed total hospital costs incurred by patients who survived and had valid cost data (referred to as the SBP_Cost_ subgroup) as a part of a subgroup analysis within the SBP_LOS_ group.

### 2.2. Independent and Dependent Variables

We stratified both the SBP_LOS_ group and the SBP_Cost_ subgroup by the timing of albumin administration from hospital admission. Prior to the analysis of data, we categorized patients by timing of albumin administration concordant with AASLD and European Association for the Study of the Liver (EASL) practice guidelines that recommend administering albumin within 1 day of SBP diagnosis. Patients who received an albumin infusion in ≤24 hours of admission were categorized as the “timely albumin” group, while those who received albumin > 24 hours after admission or no albumin at all were categorized as the “non-timely albumin” group. We combined patients who received albumin more than 24 hours after admission with those who did not receive albumin at all based on the low sample size of the no-albumin group. Additionally, preliminary analysis revealed no differences in patient demographics between the non-timely albumin and no-albumin groups. In our study, the initiation of albumin infusion occurs either within 24 hours of admission or later, thus aligning with our SBP diagnosis timeframe. We defined albumin administration as the receipt of any medication with a generic or brand name of albumin.

LOS was evaluated as the main outcome, defined as the time from hospital admission to discharge (time to discharge). The secondary outcome was total hospital costs. We derived total hospital costs from hospital charges, estimated based on a standard hospital-specific cost-to-charge ratio. We excluded hospitalizations with “missing/null” charges or missing cost-to-charge ratios for all index years from the calculation of total hospital costs. However, these hospitalizations were still included in the LOS analysis. We adjusted the hospital cost data and reported them as June 2017 dollars by using the Producer Price Index database from the Bureau of Labor Statistics [30]. We utilized a compound annual growth rate to account for missing cost data from January 2009 through May 2009, to discount the Producer Price Index from June 2009 to January 2009, and to calculate Producer Price Index from June 2009 to June 2017.

### 2.3. Statistical Analyses

We calculated descriptive statistics for baseline patient demographics (e.g., age, sex, and ethnicity) and hospital characteristics (e.g., hospital type and bed size) and summarized binary or categorical variables via counts and percentages and continuous variables via means and standard deviations. Baseline severity was calculated using the Charlson Comorbidity Index (CCI) [31]. Additionally, we performed an exploratory analysis to summarize patient demographics and clinical characteristics based on ICU admission status.

A Kaplan-Meier analysis with a log-rank test was used to examine time to hospital discharge after censoring for mortality. We also used an adjusted Cox proportional hazards regression model to observe the association between albumin infusion timing (timely vs. non-timely) and rate of hospital discharge and explore the effect of covariates. Hazard ratios with 95% confidence intervals (CIs) at a significance threshold of *p* < 0.05 were reported. A generalized linear model (GLM) specified with gamma distribution and log link function, and random effects to account for clustering of patients by hospital, was employed to examine the association between albumin infusion timing (timely vs. non-timely) and total hospital costs as a continuous outcome. We calculated adjusted parameter estimates (PEs) and log-transformed them into cost ratios with 95% CIs and a significance level of <0.05.

The Cox regression and GLM models were adjusted using various covariates, including baseline patient characteristics such as age, sex, ethnicity, admission type, payer type, year of admission, and baseline hospital characteristics such as bed size, teaching status, urban vs. rural, index year, acute vs. non-acute, and census region. We also examined the association of the outcomes of interest with important complications of cirrhotic patients: AKI severity, gastrointestinal bleeding, and hepatic encephalopathy (Supplementary Table 1). We categorized AKI severity as none (AKI stage 0), mild (AKI stage 1), and moderate/severe (AKI stages 2 and 3) using the 2012 Kidney Disease Improving Global Outcomes (KDIGO) guideline framework [32] and baseline SCr values (closest to hospital admission within a 90-day prior to index period; if unavailable, we utilized the first SCr during the visit and did not impute) (Supplementary Table 3). Similarly, we flagged patients who received renal replacement therapy (RRT) as AKI stage 3. Urine output was not used for AKI-KDIGO staging since it is unavailable within the Cerner EHR database. We also adjusted for mechanical ventilation, use of vasopressor therapy, non-selective beta-blockers, and steroids to consider essential medical interventions. Baseline values of blood glucose levels (Supplementary Table 3), Model for End-Stage Liver Disease (MELD-Na), and quick sequential organ failure assessment (qSOFA) at presentation were extracted from recorded laboratory data and served to adjust baseline severity [33–35]. SAS version 9.4 (SAS Institute Inc., Cary, NC) was used to perform all statistical analyses.

## 3. Results

### 3.1. Study Group and Patient Characteristics

We identified 339,727 hospitalizations of adult patients admitted with cirrhosis (Figure 1). Certain hospitalizations were excluded based on ineligibility due to having had a liver transplant (*n* = 13,964) or lacking necessary laboratory data (*n* = 52,049). Additionally, 199,680 hospitalizations were removed due to insufficient or weak data, for example, unknown treatment or outcome owing to transfer from another ICU (*n* = 111), outlier length of stay (*n* = 6,785), or no record of fluid resuscitation (*n* = 191,319). Overall, 1,308 hospitalizations met the selection criteria which included diagnoses of cirrhosis and SBP where patients received fluid resuscitation, ≥1 antibiotic during their visit, and survived to discharge (“SBP_LOS_” group), of which 301 hospitalizations containing valid cost data were included in the subgroup analysis for determining total costs (“SBP_Cost_” subgroup).  Table 1 shows patient characteristics for the SBP_LOS_ and SBP_Cost_ group overall and by albumin timing. We observed a total of 6 patients (0.46% of *n* = 1,308) who could have developed nosocomial SBP. There were 3 patients in each albumin group (*p* = 1.000). We identified 13 patients (0.99%) with MDRO infection in our population with 5 patients in the timely albumin group and 8 patients in the non-timely group (*p* = 0.584) (Supplementary Table 4).

### 3.2. LOS Analysis

In the overall SBP_LOS_ group, the mean (±SD) age was 57.3 ± 12.1 years, 40.0% (*n* = 523) were female, and 73.9% (*n* = 967) were Caucasian. High levels of disease severity were observed. The mean ± SD values for MELD-Na, total bilirubin, and creatinine were 22.6 ± 9.1, 6.0 ± 7.3 mg/dL, and 2.1 ± 1.9 mg/dL, respectively. Hepatic encephalopathy and gastrointestinal bleeding were common, at rates of 39.1% and 15.5%, respectively. The vast majority (86.9%) of patients were emergently hospitalized, and the mean unadjusted overall hospital LOS was 8.8 ± 7.6 days.

Out of the 1,308 cases that met the inclusion criteria, albumin was infused in ≤24 hours of admission (“timely albumin”) in less than half of the cases (47.8%, *n* = 626). Female sex, African Americans, Asian/Pacific Islanders, and Caucasians had a lower proportion of timely albumin administration, while the commercial payer group had a higher proportion compared to the non-timely group. The mean (±SD) baseline CCI and MELD-Na was higher in the timely albumin group compared to the non-timely albumin group (7.7 ± 3.3 vs. 7.6 ± 3.3 and 25.2 ± 8.8 vs. 20.2 ± 8.8, respectively). Compared to the non-timely group, the timely albumin group had lower rates of steroid use (8.1% vs. 8.9%) and non-selective beta-blocker use (22.0% vs. 28.3%), but a higher proportion of mechanical ventilation (14.1% vs. 12.5%) and vasopressor use (22.8% vs. 17.4%). A higher mean (±SD) duration of antibiotic therapy was observed in the timely albumin group vs. the non-timely albumin group (131.0 ± 227.9 vs. 128.2 ± 181.4 hours). Additional demographic and hospital characteristics for the SBP_LOS_ group are reported in Supplementary Table 5.

MELD-Na scores varied across of all four groups with highest MELD-Na scores for patients who were admitted to the ICU and those who received timely albumin (Supplementary Table 6). Mean SCr was highest among ICU patients receiving timely and non-timely albumin (2.6 mg/dL in both groups) and lowest among non-ICU patients receiving non-timely albumin (1.7 ± 1.6 mg/dL). Out of the 262 cases where vasopressors were used, 65 (24.8%) were in a non-ICU setting (e.g., emergency department and step-down unit).

Among ICU-based encounters, unadjusted hospital length of stay was shorter for those receiving timely albumin (9.5 ± 8.5 days) compared to non-timely albumin (12.3 ± 9.8 days). Total unadjusted hospital costs were also lower in the ICU care setting among the timely group ($25,530.9 ± 19,646.1) compared to the non-timely albumin ($27,124.9 ± 19,990.2).

**Figure 2:**
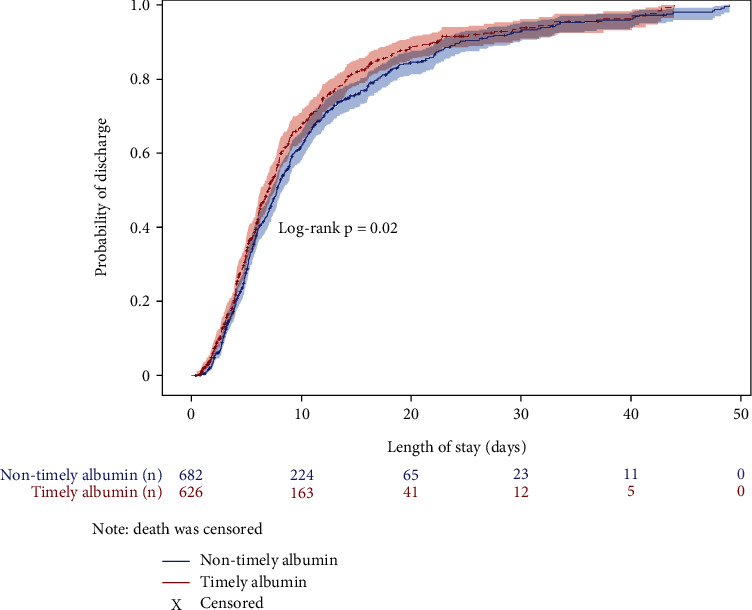
Kaplan-Meier curve showing probability of discharge in patients receiving timely vs. non-timely albumin.

The mean (±SD) unadjusted LOS was lower in patients who received timely albumin than non-timely albumin (8.1 ± 7.0 vs. 9.5 ± 8.2 days, Supplementary Figure 1). The Kaplan Meier curve depicts the probability of discharge while censoring for death (Figure 2; shaded portion indicates confidence intervals) and shows that the timely albumin group had a higher probability of being discharged alive at any point in time (log-rank *p* = 0.02). Median time to hospital discharge in the timely albumin group was 6.95 days (95% CI: 6.29 to 7.62) compared to 7.78 days (95% CI: 7.27 to 8.33) in the non-timely albumin group. An adjusted Cox proportional hazards regression model showed that rate of hospital discharge increased by 31% in the timely albumin group compared to the non-timely albumin group (HR = 1.31, 95% CI: 1.17 to 1.46, *p* < 0.001). Higher AKI severity, mechanical ventilation, hyperglycemia, and higher qSOFA significantly decreased rate of discharge (Table 2).

### 3.3. Hospital Cost Analysis

A total of 301 hospitalizations were identified to have valid cost data and comprised the SBP_Cost_ subgroup (timely albumin: *n* = 131, 43.5%; non-timely albumin: *n* = 170, 56.5%). The total mean (±SD) cost of hospitalization among all 301 cases was $16,664.2 ± 13,662.6. The mean (±SD) overall age in the SBP_Cost_ subgroup was 56.6 ± 12.8, and 40.2% were female. The rate of mechanical ventilation was 5.3% in the timely albumin group and 6.5% in the non-timely group. The mean (±SD) MELD-Na in the SBP_Cost_ subgroup was higher among those who received timely albumin compared to those who received non-timely albumin (23.3 ± 8.4 vs. 19.1 ± 8.4). Additional demographic and hospital characteristics for the SBP_Cost_ subgroup are reported in Supplementary Table 5.

The mean (±SD) unadjusted total costs were lower in the timely albumin group compared to the non-timely group ($16,349.6 ± 13,034.1 vs. $16,906.6 ± 14,161.2, Supplementary Figure 2). Risk-adjusted analysis using a GLM (Table 3) showed the timely albumin group to be significantly associated with lower hospital costs by 16% (cost ratio: 0.84, 95% CI 0.72 to 0.98, *p* = 0.027). AKI severity, gastrointestinal bleeding, mechanical ventilation, and qSOFA score of 3 remained statistically significant as predictors of increased hospital cost.

## 4. Discussion

Spontaneous bacterial peritonitis is a serious complication of decompensated cirrhosis. It predisposes patients to conditions such as hepatorenal syndrome, septic shock, and hepatic encephalopathy, often requiring multidisciplinary teams with highly specialized healthcare providers [36]. The 2009 and 2012 American Association for the Study of Liver Diseases' practice guidelines recommend that SBP be managed with administration of albumin [17, 18]. The American Association for the Study of Liver Diseases' 2021 practice guidance also recommends the use of albumin in the management of SBP to prevent AKI progression [37]. Similarly, the European Association for the Study of the Liver also recommends that all patients with SBP be treated with albumin on days one to three of hospitalization in an effort to curtail the development of hepatorenal syndrome [38].

In this study, we utilized real-world data to analyze key outcomes of hospital LOS measured as time to discharge from hospital and hospital cost among patients with decompensated cirrhosis and SBP who received antibiotic therapy and fluid resuscitation. Our study is aimed at measuring the impact of following recommended guidelines pertaining to timely albumin administration for the management of SBP. Findings indicate that timely administration of albumin could reduce the length of hospital stays, even in patients with higher degrees of severity, as measured by MELD-Na scores and CCIs. We found that patients who received albumin in ≤24 hours of hospitalization were more likely to experience shorter LOS and incur lower total hospital costs. A plausible reason for the lower hospital costs in patients receiving timely albumin infusion could be due to shorter length of hospital stay, shorter length of ICU stay, or not requiring an ICU stay, which is supported by our descriptive statistics (ICU cases receiving timely albumin had a considerably lower length of hospital stay and subsequently incurred lower costs compared to cases with non-timely albumin). However, future studies could assess the exact contribution of ICU stay to the overall LOS in timely vs. non-timely albumin patients. Notably, despite higher degrees of severity measured by MELD-Na scores and CCIs, cases of timely albumin administration experienced shorter LOS (unadjusted ~1.5 days). However, there were more cases that did not receive timely albumin (52.2%) than those that did, highlighting a potential opportunity for improvement and better guideline-directed therapy.

Management of SBP has become increasingly complex as more SBP cases are caused by gram-positive and/or MDRO, particularly in nosocomial settings [39, 40]. These infection episodes tend to be more difficult to treat and may take longer to resolve [36], predisposing patients to adverse hemodynamic and immune responses associated with systemic infection [41–43]. These patients may also suffer major complications such as AKI and hepatorenal syndrome, which are associated with a high risk of death, increased healthcare resource utilization, and longer hospitalizations [44, 45]. Our exploratory analysis showed minimal presence of MDRO-related infections, and prevalence did not differ between albumin groups. Exploratory analysis also indicated a low frequency of potential nosocomial SBP cases in our population, which might not provide sufficient statistical power to detect a significant difference in outcomes or contribute to variance in the timely and non-timely albumin groups. Additionally, our inclusion criteria requiring SBP diagnosis and antibiotic treatment within 24 hours of admission would exclude patients with nosocomial SBP from our cohort. While investigating nosocomial SBP infections was not the main focus of this study, our future work could consider incorporating nosocomial SBP infections.

Our data shows that timely albumin administration in conjunction with an antibiotic regimen may help lower hospital resource utilization, including shortened duration of hospitalization. ICU care is expensive [46], particularly in patients requiring mechanical ventilation, in part because these patients remain hospitalized significantly longer as compared to patients not requiring mechanical ventilation [47]. Although our timely albumin group showed comparable rates of mechanical ventilation and ICU admission to the non-timely albumin group, the overall time of hospital stay was shorter in the timely albumin group, which likely contributed to reductions in total hospital costs. These data are congruent with prior studies that demonstrated an overall association between albumin infusion and improved survival, lesser complications, and better management of ascites, with subsequent lower length of hospitalization among patients with end-stage liver disease [19, 20, 22, 23, 48]. While our study showed a positive correlation between patients receiving albumin within 24 hours, it is possible that these benefits could be appreciated further with even earlier administration. Ebied et al. demonstrated that albumin administered within 6 hours of SBP diagnosis contributed to a decrease in incidence of AKI and mortality rate [49].

Although the SCr in the entire study population is suggestive of underlying chronic renal impairment, the utilization of albumin corresponded with increasing disease severity when considering MELD-Na and CCI scores. This coincides with prior data that albumin treatment was most beneficial in SBP cases with high risks of mortality [50]. It is important to point out that timely albumin did not simply overcome any differences in hospital resource utilization attributable to higher levels of disease severity; it decreased both length of hospitalization and total hospital costs. The Kaplan-Meier and multivariable models show that after adjusting for variables such as disease severity and comorbidity, timely albumin was associated with 31% higher rate of discharge, reduced length of hospital stay, and 16% reduction in hospital costs. These data suggest that albumin may be underutilized in patients with cirrhosis and SBP. Early albumin administration during SBP (in patients with less acuity than observed in this study) may decrease hospital resource utilization to the extent that it is comparable to patients with minimal comorbidity and less severe hepatic decompensation. This is an essential consideration as albumin use is often denied by payers and hospital administrators who cite its high cost. Our data show that timely albumin in SBP patients may not only be cost-effective but also cost-saving in patients who may not be receiving it in current practice.

As with any retrospective database study, there are inherent limitations pertaining to data capture, one of which was albumin dosage in our study. However, future studies can focus on assessing impact of timely albumin administration based on dosing. Other limitations include lack of systematic collection of specific variables to precisely define independent and dependent variables such as ascitic fluid polymorphonuclear leukocyte counts and culture results as well as exact time stamps for ICU admission and discharge; lack of data from health encounters in outpatient settings and at non-Cerner facilities; interinstitutional variability in patient assessment, care, and adjudication of outcomes; and total subsequent volume of administered albumin/fluid. However, we were able to overcome many of the shortcomings prevalent to administrative databases as we were able to access richer clinical details such as detailed medication history and serial laboratory data as well as track individual patients longitudinally. Regarding our statistical inference, the potential for residual confounding remains; we adjusted for patient and hospital characteristics and severity indices that may resolve some concerns about exposure differences based on these factors. Being cross-sectional data, the study was not designed to establish causality; however, it does present compelling evidence to correlate timely albumin use and patient outcomes. However, future studies should be conducted to confirm this.

## 5. Conclusions

In summary, timely albumin infusion in patients with decompensated cirrhosis and SBP is associated with shorter hospital stays and reduced total hospital costs. Optimal administration of albumin may improve patient outcomes and reduce the burden of these conditions on the healthcare system, especially in patients with advanced complications of end stage liver disease. The data suggests that future research should focus on conducting large-scale, multicenter prospective trials, to evaluate the optimal dosage, duration, clinical efficacy, and cost-effectiveness of albumin in patients with cirrhosis and SBP.

## Figures and Tables

**Table 1:** Patient characteristics in the length of stay group and cost group.

Characteristic	SBP_LOS_ group			SBP_Cost_ subgroup		
	Overall	Timely albumin	Non-timely albumin	Overall	Timely albumin	Non-timely albumin
	*N* = 1,308	*N* = 626	*N* = 682	*N* = 301	*N* = 131	*N* = 170
Age (in years)						
Mean (SD)	57.3 (12.1)	57.2 (11.8)	57.5 (12.3)	56.6 (12.8)	55.8 (12.2)	57.3 (13.2)
Median (p25, p75)	57.0 (50.0, 65.0)	58.0 (50.0, 64.0)	57.0 (50.0, 65.0)	57.0 (49.0, 65.0)	57.0 (49.0, 64.0)	57.0 (49.0, 65.0)
18-29, *n* (%)	16 (1.2)	8 (1.3)	8 (1.2)	9 (3.0)	7 (5.3)	2 (1.2)
30-49, *n* (%)	288 (22.0)	140 (22.4)	148 (21.7)	72 (23.9)	31 (23.7)	41 (24.1)
50-64, *n* (%)	676 (51.7)	323 (51.6)	353 (51.8)	144 (47.8)	63 (48.1)	81 (47.6)
65+, *n* (%)	328 (25.1)	155 (24.8)	173 (25.4)	76 (25.2)	30 (22.9)	46 (27.1)
Female, *n* (%)	523 (40.0)	227 (36.3)	296 (43.4)	121 (40.2)	46 (35.1)	75 (44.1)
Ethnicity, *n* (%)						
African American	97 (7.4)	39 (6.2)	58 (8.5)	20 (6.6)	9 (6.9)	11 (6.5)
Asian/Pacific Islander	19 (1.5)	9 (1.4)	10 (1.5)	0 (0.0)	0 (0.0)	0 (0.0)
Caucasian	967 (73.9)	461 (73.6)	506 (74.2)	231 (76.7)	100 (76.3)	131 (77.1)
Hispanic	21 (1.6)	13 (2.1)	8 (1.2)	6 (2.0)	2 (1.5)	4 (2.4)
Other	193 (14.8)	98 (15.7)	95 (13.9)	44 (14.6)	20 (15.3)	24 (14.1)
Unknown	11 (0.8)	6 (1.0)	5 (0.7)	0 (0.0)	0 (0.0)	0 (0.0)
Admission type, *n* (%)						
Emergency	1,136 (86.9)	532 (85.0)	604 (88.6)	263 (87.4)	116 (88.5)	147 (86.5)
Urgent	114 (8.7)	64 (10.2)	50 (7.3)	17 (5.6)	5 (3.8)	12 (7.1)
Elective	58 (4.4)	30 (4.8)	28 (4.1)	21 (7.0)	10 (7.6)	11 (6.5)
Payer group, *n* (%)						
Medicare	450 (34.4)	206 (32.9)	244 (35.8)	121 (40.2)	50 (38.2)	71 (41.8)
Medicaid	348 (26.6)	153 (24.4)	195 (28.6)	95 (31.6)	43 (32.8)	52 (30.6)
Commercial	281 (21.5)	157 (25.1)	124 (18.2)	57 (18.9)	27 (20.6)	30 (17.6)
Other^a^	42 (3.2)	17 (2.7)	25 (3.7)	7 (2.3)	2 (1.5)	5 (2.9)
Self	64 (4.9)	36 (5.8)	28 (4.1)	11 (3.7)	6 (4.6)	5 (2.9)
Null	123 (9.4)	57 (9.1)	66 (9.7)	10 (3.3)	3 (2.3)	7 (4.1)
CCI, mean (SD)	7.6 (3.3)	7.7 (3.3)	7.6 (3.3)	7.2 (3.1)	7.1 (2.9)	7.3 (3.3)
AKI severity, *n* (%)						
Moderate/severe	300 (22.9)	159 (25.4)	141 (20.7)	62 (20.6)	33 (25.2)	29 (17.1)
Mild	377 (28.8)	182 (29.1)	195 (28.6)	83 (27.6)	37 (28.2)	46 (27.1)
None	631 (48.2)	285 (45.5)	346 (50.7)	156 (51.8)	61 (46.6)	95 (55.9)
Gastrointestinal bleeding, *n* (%)	203 (15.5)	75 (12.0)	128 (18.8)	25 (8.3)	12 (9.2)	13 (7.6)
Hepatic encephalopathy, *n* (%)	512 (39.1)	268 (42.8)	244 (35.8)	112 (37.2)	52 (39.7)	60 (35.3)
Clinical parameters, mean (SD)						
MELD-Na	22.6 (9.1)	25.2 (8.8)	20.2 (8.8)	21.1 (8.6)	23.3 (8.4)	19.4 (8.4)
Bilirubin (mg/dL)	6.0 (7.3)	6.6 (7.5)	5.5 (7.1)	6.1 (7.5)	6.6 (7.7)	5.8 (7.4)
INR	2.1 (1.3)	2.2 (1.2)	2.0 (1.4)	1.9 (1.1)	1.9 (0.7)	1.9 (1.3)
Creatinine (mg/dL)	2.1 (1.9)	2.3 (1.8)	1.9 (1.9)	1.9 (1.6)	2.0 (1.4)	1.8 (1.7)
Sodium (mEq/L)	138.1 (5.8)	137.6 (5.7)	138.6 (5.8)	137.5 (5.5)	137.0 (5.3)	138.0 (5.7)
Glycemic status, *n* (%)						
Hyperglycemia	410 (31.3)	188 (30.0)	222 (32.6)	96 (31.9)	41 (31.3)	55 (32.4)
Hypoglycemia	76 (5.8)	43 (6.9)	33 (4.8)	8 (2.7)	4 (3.1)	4 (2.4)
Both hyper- and hypoglycemia	135 (10.3)	65 (10.4)	70 (10.3)	13 (4.3)	3 (2.3)	10 (5.9)
Neither hyper- nor hypoglycemia	687 (52.5)	330 (52.7)	357 (52.3)	184 (61.1)	83 (63.4)	101 (59.4)
Process variables, *n* (%)						
Mechanical ventilation	173 (13.2)	88 (14.1)	85 (12.5)	18 (6.0)	7 (5.3)	11 (6.5)
Vasopressors^b^	262 (20.0)	143 (22.8)	119 (17.4)	34 (11.3)	18 (13.7)	16 (9.4)
Steroids^c^	112 (8.6)	51 (8.1)	61 (8.9)	29 (9.6)	13 (9.9)	16 (9.4)
Non-selective beta-blockers^d^	331 (25.3)	138 (22.0)	193 (28.3)	87 (28.9)	33 (25.2)	54 (31.8)
Renal replacement therapy	113 (8.6)	63 (10.1)	50 (7.3)	16 (5.3)	8 (6.1)	8 (4.7)
Abx duration (hours), mean (SD)	129.5 (204.2)	131.0 (227.9)	128.2 (181.4)	128.8 (316.7)	134.6 (383.9)	124.3 (254.3)
Hospital LOS (days), mean (SD)	8.8 (7.6)	8.1 (7.0)	9.5 (8.2)	8.2 (5.8)	7.5 (5.3)	8.7 (6.0)

Abx: antibiotics; AKI: acute kidney injury; CCI: Charlson Comorbidity Index; INR: international normalized ratio; LOS: length of stay; MELD-Na: Model for End-Stage Liver Disease-Sodium; SBP: spontaneous bacterial peritonitis; SD: standard deviation. ^a^Military, non-governmental organization, and work compensation payer groups. ^b^Dobutamine, dopamine, epinephrine, norepinephrine, phenylephrine, and vasopressin. ^c^Budesonide (oral or nasal), cortisone, deflazacort, dexamethasone-lidocaine, prednisolone, prednisolone ophthalmic, and prednisone. ^d^Bendroflumethiazide-nadolol, carvedilol, hydrochlorothiazide-propranolol, nadolol, and propranolol.

**Table 2:** Cox proportional hazard model showing rate of hospital discharge (*N* = 1,308).

Characteristic	Hazard ratio (95% CI)	*p* value
Timely vs. non-timely albumin	1.31 (1.17-1.46)	<0.001
AKI severity: mild vs. none	0.54 (0.44-0.66)	<0.001
AKI severity: moderate/severe vs. none	0.50 (0.40-0.63)	<0.001
Gastrointestinal bleeding	0.90 (0.76-1.08)	0.267
Hepatic encephalopathy	0.98 (0.82-1.16)	0.780
Mechanical ventilation	0.57 (0.45-0.73)	<0.001
Non-selective beta-blockers	1.23 (1.06-1.43)	0.006
Hyperglycemia and hypoglycemia		
Hyperglycemia vs. neither hypo-/hyperglycemia	0.70 (0.58-0.84)	<0.001
Hypoglycemia vs. neither hypo-/hyperglycemia	0.73 (0.48-1.11)	0.138
Both hypo-/hyperglycemia vs. neither hypo-/hyperglycemia	0.54 (0.44-0.66)	<0.001
MELD-Na	1.00 (0.99-1.00)	0.318
qSOFA		
1 vs. 0	0.56 (0.43-0.74)	<0.001
2 vs. 0	0.31 (0.23-0.42)	<0.001
3 vs. 0	0.19 (0.14-0.27)	<0.001

AKI: acute kidney injury; CI: confidence interval; MELD-Na: Model for End-Stage Liver Disease-Sodium; qSOFA: quick sequential organ failure assessment. Note: Cox proportional hazards regression model was used with adjusting standard errors for within hospital clustering. We censored for patients who died during the hospitalization. Additional covariates included in the model for adjustments: age, sex, and ethnicity of the patient; geographic region and urbanicity; bed size; teaching status of the hospital; admission type; and calendar year.

**Table 3:** Generalized linear model predictors for total hospital costs as continuous outcome (*N* = 301).

Parameters	Cost ratio (95% CI)	*p* value
Timely vs. non-timely albumin	0.84 (0.72-0.98)	0.027
AKI severity: mild vs. none	1.25 (1.04-1.50)	0.018
AKI severity: moderate/severe vs. none	1.44 (1.14-1.81)	0.002
Gastrointestinal bleeding	1.38 (1.06-1.79)	0.017
Hepatic encephalopathy	0.89 (0.77-1.04)	0.161
Mechanical ventilation	1.58 (1.11-2.24)	0.012
Non-selective beta-blockers	0.85 (0.71-1.01)	0.072
Hyperglycemia vs. neither hypo-/hyperglycemia	1.12 (0.94-1.33)	0.195
Hypoglycemia vs. neither hypo-/hyperglycemia	1.15 (0.75-1.76)	0.513
Both hypo-/hyperglycemia vs. neither hypo-/hyperglycemia	1.72 (1.11-2.67)	0.017
MELD-Na	0.99 (0.98-1.00)	0.103
qSOFA 1 vs. 0	1.09 (0.70-1.71)	0.701
qSOFA 2 vs. 0	1.46 (0.93-2.27)	0.099
qSOFA 3 vs. 0	1.93 (1.21-3.08)	0.007

AKI: acute kidney injury; CI: confidence interval; GLM: generalized linear model; MELD-Na: Model for End-Stage Liver Disease-Sodium; qSOFA: quick sequential organ failure assessment. Note: generalized linear model (specified with a gamma distribution and log link function) with random effects was used in order to account for clustering of patients by hospital. Additional covariates included in the GLM were age, sex, and ethnicity of the patient; geographic region and urbanicity; bed size; teaching status of the hospital; admission type; and calendar year.

## Data Availability

The datasets analyzed during the current study are available from the corresponding author upon reasonable request.

## Abbreviations

AASLD: American Association for the Study of Liver Diseases
AKI: Acute kidney injury
CCI: Charlson Comorbidity Index
CI: Confidence interval
EASL: European Association for the Study of the Liver
EHR: Electronic health record
GLM: Generalized linear model
ICD: International Classification of Disease
KDIGO: Kidney Disease Improving Global Outcomes
LOS: Length of stay
MDRO: Multi-drug-resistant organisms
MELD: Model for end-stage liver disease
PE: Parameter estimate
qSOFA: Quick sequential organ failure assessment
RRT: Renal replacement therapy
SBP: Spontaneous bacterial peritonitis
SCr: Serum creatinine
STROBE: Strengthening the reporting of observational studies in epidemiology.

## Supplementary Materials

Supplementary 1 Supplementary Table 1: ICD/CPT codes for decompensated cirrhosis and cirrhosis-related complications and procedures. Supplementary Table 2: antibiotics included in the analysis. Supplementary Table 3: lab procedures for serum creatinine and glucose levels. Supplementary Table 4: prevalence of potential nosocomial SBP and MDRO in timely and non-timely albumin groups. Supplementary Table 5: additional patient demographics and hospital characteristics in length of stay and cost groups. Supplementary Table 6: patient and clinical characteristics by intensive care unit status and timing of albumin administration in the length of stay group. Supplementary Figure 1: box-and-whisker plot for length of stay—comparing treatment groups (timely versus non-timely albumin) to unadjusted length of stay in days (*N* = 1,308). Supplementary Figure 2: box-and-whisker plot for hospital costs—comparing treatment groups (timely versus non-timely albumin) to unadjusted total hospital costs in US dollars (*N* = 301).

Supplementary 2 STROBE checklist for observational studies.
