# Supplementary material for: Timely Albumin Infusion May Improve Resource Utilization in Patients with Cirrhosis and Spontaneous Bacterial Peritonitis
Source: Biomed Res Int. 2024 Jun 8;2024:6673823. doi: 10.1155/2024/6673823 (PMC11186688; doi:10.1155/2024/6673823)
Supplement: Supplementary 1 — Supplementary Table 1: ICD/CPT codes for decompensated cirrhosis and cirrhosis-related complications and procedures. Supplementary Table 2: antibiotics included in the analysis. Supplementary Table 3: lab procedures for serum creatinine and glucose levels. Supplementary Table 4: prevalence of potential nosocomial SBP and MDRO in timely and non-timely albumin groups. Supplementary Table 5: additional patient demographics and hospital characteristics in length of stay and cost groups. Supplementary Table 6: patient and clinical characteristics by intensive care unit status and timing of albumin administration in the length of stay group. Supplementary Figure 1: box-and-whisker plot for length of stay—comparing treatment groups (timely versus non-timely albumin) to unadjusted length of stay in days (N = 1,308). Supplementary Figure 2: box-and-whisker plot for hospital costs—comparing treatment groups (timely versus non-timely albumin) to unadjusted total hospital costs in US dollars (N = 301). [file 6673823.f1.docx]

**Supplementary File 1**

**Supplementary Table 1. ICD/CPT codes for decompensated cirrhosis and cirrhosis-related complications and procedures**

| **Description** | **ICD-9 Codes** | **ICD-10 Codes** | **CPT Codes** |
| --- | --- | --- | --- |
| Decompensated cirrhosis | 42.91, 44.91, 54.91, 96.06, 155.0, 155.1, 155.2, 456.0, 456.20, 571.2, 571.5, 572.2, 572.3, 572.4, 572.8, 789.5, 789.59 | C22.0, C22.1, C22.2, C22.7, C22.8, C22.9, K70.30, K72.09, K72.10, K72.90, K72.91, K74.0, K74.60, K74.69, K76.6, K76.7, I85.01, I85.11, R18.8, 06L30CZ, 06L30DZ, 06L30ZZ, 06L33CZ, 06L33DZ, 06L33ZZ, 06L34CZ, 06L34DZ, 06L34ZZ, 06L20ZZ, 06L23ZZ, 06L24ZZ, 0DL57DZ, 0DL58DZ, 0D9S30Z, 0D9S3ZZ, 0D9S40Z, 0D9S4ZZ, 0D9T30Z, 0D9T3ZZ, 0D9T40Z, 0D9T4ZZ, 0D9V30Z, 0D9V3ZZ, 0D9V40Z, 0D9V4ZZ, 0D9W30Z, 0D9W3ZZ, 0D9W40Z, 0D9W4ZZ, 0W9F30Z, 0W9F3ZZ, 0W9F40Z, 0W9F4ZZ, 0W9G30Z, 0W9G3ZZ | 37140, 37160, 37180, 37181 37182, 37183, 43204, 43205, 43243, 43244, 43400, 43401, 49080, 49081 |
| Renal replacement therapy | 38.95, 39.95, 54.98 | 3E1M39Z, 5A1D00Z, 5A1D60Z |  |
| Large volume paracentesis | 54.91 | 0W9G3ZZ |  |
| Spontaneous bacterial peritonitis | 567.23 | K65.2 |  |
| Hepatic encephalopathy | 572.2 | K72.90, K72.91, K70.41, K72.01, K72.11 |  |
| Gastrointestinal bleeding | 456.0, 530.82, 531.40, 532.00, 578.0, 578.1, 578.9 | I85.01, K92.0, K92.1, K92.2 |  |
| Mechanical ventilation | 93.90, 96.70, 96.71, 96.72 | 5A09357, 5A09457, 5A09557, 5A1935Z, 5A1945Z, 5A1955Z |  |

ICD, International Classification of Diseases; CPT, Common Procedural Terminology

**Supplementary Table 2. Antibiotics included in analysis**

| **Antibiotic classes from Veteran Affairs National Formulary List** |
| --- |
| Penicillin-g related penicillins |
| Penicillins, amino derivatives |
| Penicillinase-resistant penicillins |
| Extended spectrum penicillins |
| Cephalosporin, 1st generation |
| Cephalosporin, 2nd generation |
| Cephalosporin, 3rd generation |
| Cephalosporin, 4th generation |
| Beta-lactams antimicrobials, other |
| Chloramphenicol |
| Erythromycins/macrolides |
| Tetracyclines |
| Aminoglycosides |
| Lincomycins |
| Quinolones |
| Antituberculars |
| Methenamine salts antimicrobials |
| Nitrofurans antimicrobials |
| Sulfonamide/related antimicrobials |
| Anti-infectives, other |

Note: About 750 antibiotics were reviewed for visit counts containing above VA classes with following routes: oral, oral and topical, compounding, injectable, intravenous, intramuscular, ophthalmic, rectal, implant, and inhalation.

**Supplementary Table 3. Lab procedures for serum creatinine and glucose levels^33-35^**

| **Lab Procedure Group** | **LOINC Code** |
| --- | --- |
| Creatinine Test | 2160-0, 16189-3, 59826-8, 38483-4 |
| Glucose Test | 10449-7, 12639-1, 14743-9, 14771-0, 1521-4, 1558-6, 21309-0, 2339-0, 2340-8, 2341-6, 2345-7, 39480-9, 39481-7, 40003-6, 40042-4, 41651-1, 41652-9, 41653-7, 51596-5, 5914-7, 72516-8 |

LOINC, Logical Observation Identifiers Names and Codes

**Supplementary Table 4. Prevalence of potential nosocomial SBP and MDRO in timely and non-timely albumin group**

| **Prevalence, n** | **Timely Albumin**  **N = 626** | **Non-Timely Albumin**  **N = 682** | **p-value*** |
| --- | --- | --- | --- |
| **Nosocomial SBP**  Yes  No | 3  623 | 3  679 | 1.000 |
| **MDRO**  Yes  No | 5  621 | 8  674 | 0.584 |

MDRO, multidrug-resistant organisms; SBP, spontaneous bacterial peritonitis

*Using Fisher's Exact Test

**Supplementary Table 5. Additional patient demographics and hospital characteristics in length of stay and cost groups**

|  | SBP_LOS_ group | | | SBP_Cost_ group | | |
| --- | --- | --- | --- | --- | --- | --- |
|  | **Overall** | **Timely Albumin** | **Non-Timely Albumin** | **Overall** | **Timely Albumin** | **Non-Timely Albumin** |
|  | **N = 1,308** | **N = 626** | **N = 682** | **N = 301** | **N = 131** | **N = 170** |
| Discharge status, n (%) |  |  |  |  |  |  |
| Death | 252 (19.3) | 135 (21.6) | 117 (17.2) | 169 (56.1) | 80 (61.1) | 89 (52.4) |
| Home | 605 (46.3) | 280 (44.7) | 325 (47.7) | 39 (13.0) | 18 (13.7) | 21 (12.4) |
| Hospice | 125 (9.6) | 58 (9.3) | 67 (9.8) | 8 (2.7) | 4 (3.1) | 4 (2.4) |
| Inpatient hospital | 31 (2.4) | 21 (3.4) | 10 (1.5) | 35 (11.6) | 13 (9.9) | 22 (12.9) |
| Other post-acute care setting | 93 (7.1) | 38 (6.1) | 55 (8.1) | 43 (14.3) | 14 (10.7) | 29 (17.1) |
| Skilled nursing facility | 117 (8.9) | 40 (6.4) | 77 (11.3) | 7 (2.3) | 2 (1.5) | 5 (2.9) |
| Not specified | 85 (6.5) | 54 (8.6) | 31 (4.5) | 169 (56.1) | 80 (61.1) | 89 (52.4) |
| Index year, n (%) |  |  |  |  |  |  |
| 2009 | 20 (1.5) | 5 (0.8) | 15 (2.2) | 9 (3.0) | 2 (1.5) | 7 (4.1) |
| 2010 | 40 (3.1) | 19 (3.0) | 21 (3.1) | 19 (6.3) | 8 (6.1) | 11 (6.5) |
| 2011 | 51 (3.9) | 21 (3.4) | 30 (4.4) | 16 (5.3) | 4 (3.1) | 12 (7.1) |
| 2012 | 89 (6.8) | 36 (5.8) | 53 (7.8) | 12 (4.0) | 5 (3.8) | 7 (4.1) |
| 2013 | 127 (9.7) | 60 (9.6) | 67 (9.8) | 18 (6.0) | 9 (6.9) | 9 (5.3) |
| 2014 | 236 (18.0) | 120 (19.2) | 116 (17.0) | 26 (8.6) | 14 (10.7) | 12 (7.1) |
| 2015 | 196 (15.0) | 108 (17.3) | 88 (12.9) | 32 (10.6) | 18 (13.7) | 14 (8.2) |
| 2016 | 235 (18.0) | 117 (18.7) | 118 (17.3) | 61 (20.3) | 29 (22.1) | 32 (18.8) |
| 2017 | 231 (17.7) | 103 (16.5) | 128 (18.8) | 80 (26.6) | 31 (23.7) | 49 (28.8) |
| 2018 | 83 (6.3) | 37 (5.9) | 46 (6.7) | 28 (9.3) | 11 (8.4) | 17 (10.0) |
| Bed size, n (%) |  |  |  |  |  |  |
| 0-99 | 97 (7.4) | 38 (6.1) | 59 (8.7) | 26 (8.6) | 12 (9.2) | 14 (8.2) |
| 100-199 | 238 (18.2) | 97 (15.5) | 141 (20.7) | 92 (30.6) | 35 (26.7) | 57 (33.5) |
| 200-299 | 325 (24.8) | 156 (24.9) | 169 (24.8) | 109 (36.2) | 51 (38.9) | 58 (34.1) |
| 300-499 | 246 (18.8) | 125 (20.0) | 121 (17.7) | 51 (16.9) | 26 (19.8) | 25 (14.7) |
| 500+ | 402 (30.7) | 210 (33.5) | 192 (28.2) | 23 (7.6) | 7 (5.3) | 16 (9.4) |
| Teaching facility, n (%) | 880 (67.3) | 438 (70.0) | 442 (64.8) | 165 (54.8) | 76 (58.0) | 89 (52.4) |
| Hospital type, n (%) |  |  |  |  |  |  |
| Urban | 1,098 (83.9) | 518 (82.7) | 580 (85.0) | 256 (85.0) | 109 (83.2) | 147 (86.5) |
| Rural | 210 (16.1) | 108 (17.3) | 102 (15.0) | 45 (15.0) | 22 (16.8) | 23 (13.5) |
| Census region, n (%) |  |  |  |  |  |  |
| Midwest | 140 (10.7) | 63 (10.1) | 77 (11.3) | 44 (14.6) | 20 (15.3) | 24 (14.1) |
| Northeast | 430 (32.9) | 204 (32.6) | 226 (33.1) | 75 (24.9) | 33 (25.2) | 42 (24.7) |
| South | 462 (35.3) | 200 (31.9) | 262 (38.4) | 133 (44.2) | 49 (37.4) | 84 (49.4) |
| West | 276 (21.1) | 159 (25.4) | 117 (17.2) | 49 (16.3) | 29 (22.1) | 20 (11.8) |

LOS, length of stay; SBP, spontaneous bacterial peritonitis

**Supplementary Table 6. Patient and clinical characteristics by intensive care unit status and timing of albumin administration in the length of stay group**

|  | **ICU** | | **Non-ICU** | |
| --- | --- | --- | --- | --- |
|  | **Timely Albumin** | **Non-Timely Albumin** | **Timely Albumin** | **Non-Timely Albumin** |
|  | **N = 219** | **N = 226** | **N = 407** | **N = 456** |
| **Age (in years)** |  |  |  |  |
| Mean ± SD | 57.3 ± 12.3 | 57.9 ± 11.7 | 57.2 ± 11.6 | 57.3 ± 12.6 |
| Median (25^th^ percentile, 75^th^ percentile) | 57.0 (50.0, 65.0) | 57.0 (50.0, 64.0) | 58.0 (50.0, 64.0) | 57.0 (50.0, 65.0) |
| 18-29, n (%) | 4 (1.8) | 1 (0.4) | 4 (1.0) | 7 (1.5) |
| 30-49, n (%) | 49 (22.4) | 53 (23.5) | 91 (22.4) | 95 (20.8) |
| 50-64, n (%) | 107 (48.9) | 116 (51.3) | 216 (53.1) | 237 (52.0) |
| 65+, n (%) | 59 (26.9) | 56 (24.8) | 96 (23.6) | 117 (25.7) |
| **Female, n (%)** | 87 (39.7) | 95 (42.0) | 140 (34.4) | 201 (44.1) |
| **Ethnicity, n (%)** |  |  |  |  |
| African American | 21 (9.6) | 23 (10.2) | 18 (4.4) | 35 (7.7) |
| Asian/Pacific Islander | 1 (0.5) | 3 (1.3) | 8 (2.0) | 7 (1.5) |
| Caucasian | 149 (68.0) | 169 (74.8) | 312 (76.7) | 337 (73.9) |
| Hispanic | 2 (0.9) | 1 (0.4) | 11 (2.7) | 7 (1.5) |
| Other | 43 (19.6) | 29 (12.8) | 55 (13.5) | 66 (14.5) |
| Unknown | 3 (1.4) | 1 (0.4) | 3 (0.7) | 4 (0.9) |
| **Admission type, n (%)** |  |  |  |  |
| Emergency | 196 (89.5) | 196 (86.7) | 336 (82.6) | 408 (89.5) |
| Urgent | 15 (6.8) | 19 (8.4) | 49 (12.0) | 31 (6.8) |
| Elective | 8 (3.7) | 11 (4.9) | 22 (5.4) | 17 (3.7) |
| **Payer group, n (%)** |  |  |  |  |
| Medicare | 77 (35.2) | 83 (36.7) | 129 (31.7) | 161 (35.3) |
| Medicaid | 61 (27.9) | 55 (24.3) | 92 (22.6) | 140 (30.7) |
| Commercial | 55 (25.1) | 40 (17.7) | 102 (25.1) | 84 (18.4) |
| Other^a^ | 5 (2.3) | 9 (4.0) | 12 (2.9) | 16 (3.5) |
| Self | 8 (3.7) | 14 (6.2) | 28 (6.9) | 14 (3.1) |
| Null | 13 (5.9) | 25 (11.1) | 44 (10.8) | 41 (9.0) |
| **CCI, mean ± SD** | 7.8 ± 3.2 | 8.0 ± 3.3 | 7.6 ± 3.4 | 7.4 ± 3.3 |
| **AKI severity, n (%)** |  |  |  |  |
| Moderate/Severe | 78 (35.6) | 75 (33.2) | 81 (19.9) | 66 (14.5) |
| Mild | 83 (37.9) | 81 (35.8) | 99 (24.3) | 114 (25.0) |
| None | 58 (26.5) | 70 (31.0) | 227 (55.8) | 276 (60.5) |
| **Gastrointestinal bleeding, n (%)** | 38 (17.4) | 67 (29.6) | 37 (9.1) | 61 (13.4) |
| **Hepatic encephalopathy, n (%)** | 101 (46.1) | 94 (41.6) | 167 (41.0) | 150 (32.9) |
| **Clinical Parameters, mean ± SD** |  |  |  |  |
| MELD-Na | 27.6 ± 8.1 | 22.5 ± 9.3 | 24.0 ± 8.8 | 19.1 ± 8.3 |
| Bilirubin, mg/dL | 7.3 ± 7.3 | 5.8 ± 7.3 | 6.4 ± 7.6 | 5.4 ± 7.1 |
| INR | 2.3 ± 1.3 | 2.3 ± 1.9 | 2.1 ± 1.2 | 1.9 ± 1.2 |
| Creatinine, mg/dL | 2.6 ± 2.0 | 2.6 ± 2.5 | 2.1 ± 1.7 | 1.7 ± 1.6 |
| Sodium, mEq/L | 139.6 ± 6.5 | 140.2 ± 6.7 | 136.9 ± 5.3 | 138.0 ± 5.3 |
| **Glycemic status, n (%)** |  |  |  |  |
| Hyperglycemia | 79 (36.1) | 86 (38.1) | 109 (26.8) | 136 (29.8) |
| Hypoglycemia | 23 (10.5) | 16 (7.1) | 20 (4.9) | 17 (3.7) |
| Both hyper- and hypoglycemia | 37 (16.9) | 41 (18.1) | 28 (6.9) | 29 (6.4) |
| Neither hyper- nor hypoglycemia | 80 (36.5) | 83 (36.7) | 250 (61.4) | 274 (60.1) |
| **Process variables, n (%)** |  |  |  |  |
| Mechanical Ventilation | 67 (30.6) | 67 (29.6) | 21 (5.2) | 18 (3.9) |
| Vasopressors^b^ | 106 (48.4) | 91 (40.3) | 37 (9.1) | 28 (6.1) |
| Steroids^c^ | 20 (9.1) | 15 (6.6) | 31 (7.6) | 46 (10.1) |
| Non-selective beta blockers^d^ | 40 (18.3) | 53 (23.5) | 98 (24.1) | 140 (30.7) |
| Renal replacement therapy | 31 (14.2) | 30 (13.3) | 32 (7.9) | 20 (4.4) |
| **Antibiotic duration, hours, mean ± SD** | 135.9 ± 140.4 | 151.2 ± 167.2 | 129.1 ± 252.9 | 119.7 ± 185.9 |
| **Sepsis, n (%)** | 129 (58.9) | 99 (43.8) | 56 (13.8) | 50 (11.0) |
| **Hospital LOS, days, mean ± SD** | 9.5 ± 8.5 | 12.3 ± 9.8 | 7.3 ± 5.9 | 8.2 ± 6.8 |
| **Total hospital costs ($), mean ± SD** | 25530.9 ± 19646.1 | 27124.9 ± 19990.2 | 14659.2 ± 12045.8 | 13904.5 ± 11802.8 |

AKI, Acute Kidney Injury; CCI, Charlson Comorbidity Index; ICU, intensive care unit; INR, International Normalized Ratio; LOS, length of stay; MELD-Na, Model for End-Stage Liver Disease-Sodium; SD, standard deviation

^a^ includes military, non-governmental organization and work compensation payer groups

^b^ includes dobutamine, dopamine, epinephrine, norepinephrine, phenylephrine, vasopressin

^c^ includes budesonide (oral or nasal), cortisone, deflazacort, dexamethasone-lidocaine, prednisolone, ophthalmic prednisolone, prednisone

^d^ includes bendroflumethiazide-nadolol, carvedilol, hydrochlorothiazide-propranolol, nadolol, propranolol

**Supplementary Figure 1. Box-and-whisker plot for length of stay: comparing treatment groups (timely versus non-timely albumin) to unadjusted length of stay in days (N=1,308)**


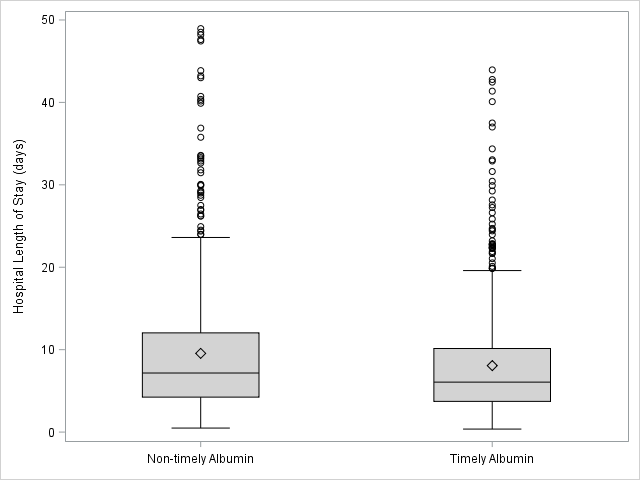

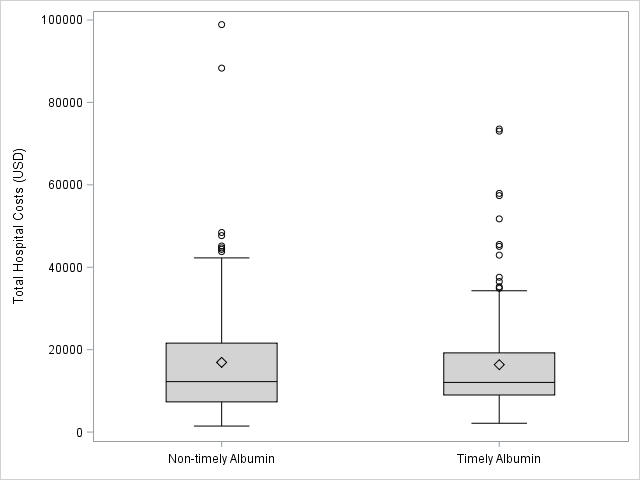


Note: Diamond represents mean; horizontal line indicates median and upper and lower margins of box indicate 25th and 75th percentile, respectively; whiskers represent range; circles represent outliers

**Supplementary Figure 2. Box-and-whisker plot for hospital costs: comparing treatment groups (timely versus non-timely albumin) to unadjusted total hospital costs in US Dollars (N=301)**


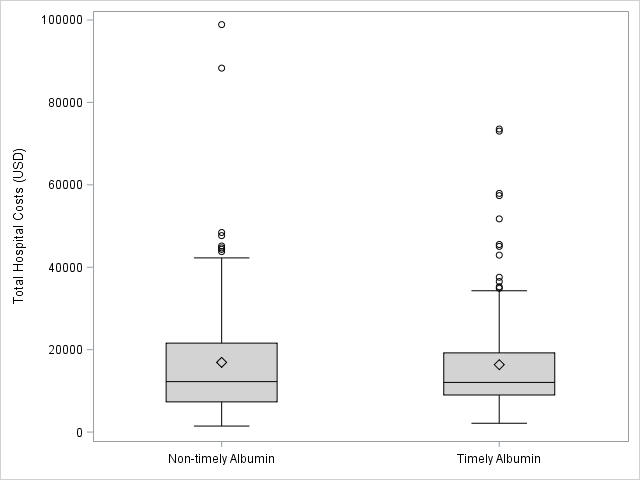


Note: Diamond represents mean; horizontal line indicates median and upper and lower margins of box indicate 25th and 75th percentile, respectively; whiskers represent range; circles represent outliers
